# Two Faces of Heme Catabolic Pathway in Newborns: A Potential Role of Bilirubin and Carbon Monoxide in Neonatal Inflammatory Diseases

**DOI:** 10.1155/2020/7140496

**Published:** 2020-08-18

**Authors:** Wiktoria Osiak, Sławomir Wątroba, Lucyna Kapka-Skrzypczak, Jacek Kurzepa

**Affiliations:** ^1^Department of Medical Chemistry, Medical University of Lublin, 20-093, Poland; ^2^Neonatal Department, Independent Public Health Care Facility, Puławy 24-100, Poland; ^3^Department of Molecular Biology and Translational Research, Institute of Rural Health, Jaczewskiego 2, 20-090 Lublin, Poland

## Abstract

In an infant's body, all the systems undergo significant changes in order to adapt to the new, extrauterine environment and challenges which it poses. Fragile homeostasis can be easily disrupted as the defensive mechanisms are yet imperfect. The activity of antioxidant enzymes, i.e., superoxide dismutase, catalase, and glutathione peroxidase, is low; therefore, neonates are especially vulnerable to oxidative stress. Free radical burden significantly contributes to neonatal illnesses such as sepsis, retinopathy of premature, necrotizing enterocolitis, bronchopulmonary dysplasia, or leukomalacia. However, newborns have an important ally—an inducible heme oxygenase-1 (HO-1) which expression rises rapidly in response to stress stimuli. HO-1 activity leads to production of carbon monoxide (CO), free iron ion, and biliverdin; the latter is promptly reduced to bilirubin. Although CO and bilirubin used to be considered noxious by-products, new interesting properties of those compounds are being revealed. Bilirubin proved to be an efficient free radicals scavenger and modulator of immune responses. CO affects a vast range of processes such as vasodilatation, platelet aggregation, and inflammatory reactions. Recently, developed nanoparticles consisting of PEGylated bilirubin as well as several kinds of molecules releasing CO have been successfully tested on animal models of inflammatory diseases. This paper focuses on the role of heme metabolites and their potential utility in prevention and treatment of neonatal diseases.

## 1. Introduction

A healthy infant is born with a concentration of bilirubin below 5 mg/dl [1]; then, serum bilirubin concentration rises quickly, up to 12 mg/dl within first 4-5 days. In pathological conditions, the growth is even more accelerated and serum bilirubin concentration, in the absence of appropriate treatment, may rise even above 40 mg/dl. The rapid increase in bilirubin appears at newborns due to both intense hemolysis and insufficiency of a liver enzyme, uridine 5′-diphospho-glucuronosyltransferase (UGT), involved in bilirubin catabolism. The increased hemolysis occurs as fetal hemoglobin (HbF), which is produced by erythroid precursor cells from 10-12 week of pregnancy till birth [2], switches to adult hemoglobin A (HbA) soon after the labor. Within the perinatal period, HbF is intensively eliminated and exchanged by HbA, leading to the low HbF concentration observed in adults (around 1% of total Hb) [3]. The increased hemoglobin catabolism is consistent with increased degradation of heme. Although this process is physiologically justified because unique properties of HbF only function during prenatal period, the mechanism of gene silencing responsible for the reduced synthesis of HbF is not fully understood [4].

Previously, some researchers have wondered whether the observed increase in bilirubin is only a side effect of the metabolic changes at newborn infants or such growth brings specific benefits [5]. Bilirubin is a compound with known antioxidant properties [6–8]; therefore, an increase in its concentration may be necessary to maintain a proper oxidative balance in the perinatal period or maybe not bilirubin but another compound formed in the heme catabolic pathway—CO is the hero of the perinatal period? This review work explores both of these options.

## 2. Heme Functions and Its Catabolic Pathway

Heme moiety, consisting of an iron ion and porphyrin, plays a crucial role as a prosthetic group of apo-heme proteins, i.e., hemoglobin, myoglobin, catalase, guanylate cyclase, and cytochromes. All of them perform important functions participating in oxygen transportation, electron transfer reactions, and catalysis [9]. Heme is also a regulator of gene expression. Bach1 and Bach2 are transcriptional suppressors involved in heme metabolism, cell cycle, oxidative stress response, and immunity [10, 11]. Heme binding to Bach1 and Bach2 inhibits their DNA-binding activity, which results in inter alia, heme oxygenase 1 induction. Heme is also a ligand for nuclear hormone receptors, REV-ERB*α* and REV-ERB*β*, involved in various biological processes including lipid and carbohydrate metabolism, cell differentiation, and circadian rhythms [12, 13]. Other target molecules for heme moiety are PAS domain of circadian factor period 2 (Per2) and neuronal PAS protein 2 (NPAS2)—transcription activator proteins associated with circadian rhythms [13]. Heme-regulated inhibitor kinase (HRI) is activated or inactivated depending on heme concentration. When heme is deficient, activated HRI phosphorylates eukaryotic translational initiation factor 2 (eIF2*α*) which leads to downregulation of *α*- and *β*-globin translation. This mechanism provides balanced synthesis of hemoglobin [14].

During intravascular hemolysis, a vast amount of hemoglobin is released into circulation. Haptoglobin (Hp), an acute phase protein, binds to hemoglobin and creates complexes which are subsequently taken up by hepatocytes and macrophages of the reticuloendothelial system via the CD163 receptor. Once binding capacity of Hp is saturated, free Hb is oxidized to methemoglobin and releases heme [15, 16]. In spite of many important biological functions mentioned above, a free heme molecule embodies a threat and acts in a destructive way [15]. Due to its lipophilic properties, heme intercalates in the membranes and modifies cellular structures [17]. Heme activates inflammatory reactions as it induces expression of adhesion molecules—intercellular adhesion molecule-1 (ICAM-1) and vascular cell adhesion molecule-1 (VCAM-1) through the signaling pathway of transcription factors nuclear factor-kappa beta (NF-*κ*B) [18]—and stimulates neutrophils through protein kinase C [19]. Heme overload leads to intensified production of reactive oxygen species (ROS) and tumor necrosis factor (TNF) [19]. Hemin, an oxidized form of heme, also exerts proinflammatory and prooxidant effects [20]. In brief, free heme is too toxic to remain “at large” in plasma or cells. Indeed, free heme in plasma is promptly scavenged and neutralized by hemopexin [21]. Afterward, heme-hemopexin complexes are cleared from circulation by hepatocytes and macrophages via the CD91 receptor and undergo lysosomal degradation [15, 16, 21].

The intracellular enzyme, named heme oxygenase (HO), which initiates degradation of free heme was discovered in the 1970s. To date, two isoforms of HO were described: HO-1 and HO-2. Constitutively expressed HO-2 is synthesized mainly in the brain, testes, and vascular system [22–25]. Under normal conditions, HO-1 is expressed at high level in the spleen and reticuloendothelial cells involved in red blood cell degradation [25]. HO-1 has attracted researchers' attention as it is obviously upregulated in response to oxidative stress. The list of HO-1 inducers is long and includes, e.g., free heme molecules, hemin, metals, cytokines, vasoactive compounds, nephrotoxin, and endotoxin [26]. Enzymatic oxidative ring cleavage of heme molecule results in formation of CO, Fe^2+^, NADP^+^, and biliverdin according to the reaction presented below (Equation (1)):

(1) $$\begin{array}{c} \text{Heme}+\text{NADPH}+{\text{H}}^{+}+3{\text{O}}_{2}⟶\text{biliverdin}+{\text{NADP}}^{+}+{\text{Fe}}^{2+}+\text{CO}+{\text{H}}_{2}\text{O} \end{array}$$

The carbon atom in the carbon monoxide molecule is detached directly from the heme ring during its cleavage. A free iron ion, which is released as one of the products of the reaction, is able to react with hydrogen peroxide and yield hydroxyl radicals; therefore, it can cause oxidative damage. However, it is noteworthy that induction of HO-1 is followed by upregulation of ferritin which binds free iron and neutralizes its cytotoxic effect [15].

There are several identified types of HO-1 gene polymorphisms, among which one is especially interesting in terms of potential clinical significance. The number of (GT)n dinucleotide repeats in 5-flanking regions of HO-1 gene varies between 12 and 40 [27, 28]. This polymorphism of the promoter region was found to affect the level of HO-1 expression in response to stimuli. In adults, shorter variant with fewer than 25 repeats was associated with higher HO-1 expression and better resistance to oxidative stress-related diseases according to some reports [27–29]. Obese children aged 6-17 with longer GT repeats in the HO-1 gene promoters were more susceptible to nonalcoholic fatty liver disease [30]. It might seem that fewer GT repeats in newborns should be associated with more severe jaundice and higher probability of phototherapy. Indeed, some researches confirm this hypothesis [31–33]. However, other published studies contradict the effect of HO-1 polymorphism on total serum bilirubin level [28, 34, 35]. One of the studies points out that polymorphism of HO-1 gene promoter can be an underlying cause of prolonged jaundice at breast-fed babies [28]. These discrepancies might be connected with different definitions of short length promoter variant and differences between various ethnic groups, then require further investigation [27].

The characteristic feature of HO-1 expression in newborns is that it rises for the first three days following labor and afterward it decreases [36]. On day 5, the expression is at the same level as at the moment of birth and then it continues to drop. HO-1 mRNA content is higher in premature newborns in comparison with term infants but with the same rise-drop pattern [36]. The changes in HO-1 expression at the early stage of life are reflected by bilirubin concentration which increases within first days of life and then it gradually declines.

## 3. Is Bilirubin Only a Toxic Waste Product?

**Figure 1:**
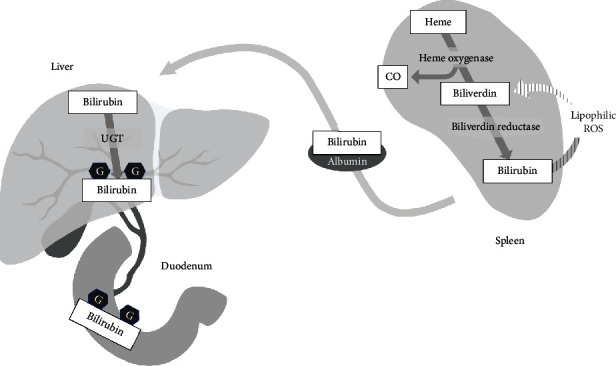
Bilirubin is formed during the degradation of heme by two forms of heme oxygenases (HO-1 and HO-2). HO-2 is constitutively expressed in various tissues. Under normal conditions HO-1 is expressed in selected organs, e.g., the spleen. The products of the reaction are green biliverdin and carbon monoxide (CO). Next, biliverdin undergoes reduction to yellow bilirubin. In the presence of lipophilic ROS, bilirubin can be reversely converted to biliverdin. Water-insoluble bilirubin is bound to serum albumin (1 g of albumin binds to 8 mg of bilirubin [38]) and transported to the liver, where glucuronidation takes place. Finally, conjugated bilirubin is excreted with the bile. G: glucuronide; UGT: 5′-diphospho-glucuronosyltransferase.

At first, bilirubin was considered only a useless outcome of heme degradation. However, bilirubin is not the best candidate for pure waste end-product. It is toxic in high concentration and its synthesis by biliverdin reductase depends on nicotinamide adenine dinucleotide phosphate (NADPH). Furthermore, bilirubin neutralization and elimination is energy-consuming and requires additional processes such as glucuronidation (Figure 1). Nevertheless, most of the mammals and some other vertebrates excrete bilirubin as the main end-product of heme catabolism. Among the mammals, biliverdin is excreted as the only bile pigment by sloths and anteaters and as the principal end-product by, e.g., a nutria (90%) and a rabbit (60-70%) [37]. In spite of the lack of uniformity in the world of animals, it can be assumed that biliverdin reductase evolved because it brought some benefits to the organism.

In fact, thorough research focused on the compound brought to light interesting data. In 1954, Karl Bernhard published a report in which he declared that bilirubin possesses antioxidant properties which protect vitamin A and unsaturated fatty acids against oxidation [39]. Stocker et al. in 1987 proved that *in vitro* bilirubin's ability to scavenge peroxyl radicals is even more prominent than the ability of another powerful antioxidant—*α*-tocopherol [6]. To date, there were many studies examining bilirubin's activity *in vitro*, but also considerable effort has been made to confirm antioxidant properties of bilirubin *in vivo.* Nonetheless, the significance of neonatal hyperbilirubinemia is still not fully understood and requires further investigation because bilirubin might turn out to be a potent ally to newborns in their first days of life.

Pulmonary respiration after birth exposes newborns to significant oxidative stress resulting from a sharp increase of partial oxygen pressure in bodily tissues. Initially, neonatal antioxidant defense is not fully mature, especially in preterm infants. Apparently, activity of the most important antioxidant enzymes—superoxide dismutase (SOD), catalase (CAT), and glutathione peroxidase (GPx)—increases in the fetus at the last weeks of pregnancy [40] (it is significantly higher at term neonates). Lack of prooxidant/antioxidant balance results in overproduction of free radicals and damage to cellular proteins, lipids, and DNA. Malondialdehyde (MDA), a by-product of lipid peroxidation of polyunsaturated fatty acids, is an acknowledged marker of oxidative stress which was scrutinized in several researches over neonatal hyperbilirubinemia. Some studies reported lower concentration of MDA in babies with high bilirubin concentration of 20 mg/dl [41] or even 25 mg/dl [42]. Other studies declared contrary results, suggesting that this issue is still unsolved [43, 44]. Researches focusing on the influence of bilirubin on total plasma antioxidant capacity (TPAC) also give inconsistent outcomes [45]. It should be remembered that bilirubin is just one of many parameters affecting the amount of TPAC. However, in most studies concerning term neonates, TPAC seems to increase in the presence of high bilirubin concentration [7, 42, 46].

During cell culture studies, the researchers noted that a nanomolar bilirubin concentration was sufficient to control high oxidative stress caused by hydrogen peroxide [47]. There is a hypothesis saying that bilirubin turns back into biliverdin when is oxidized by hydrogen peroxide (Figure 1) [48]. At this reaction, there is probably no additional enzyme involved, unlike in glutathione cycle. Subsequently, the biliverdin reductase collaborates with its reducing cofactor, NADPH, to recoup a pool of bilirubin. Existence of bilirubin/biliverdin catabolic cycle might be a compromise which would allow to take an advantage of strong antioxidant properties of bilirubin while reducing the risk of cytotoxicity [49]. According to some researches, it would explain how such a little intracellular concentration of bilirubin (below 10 nM) could protect membrane lipids and membrane proteins against peroxidation [48]. However, a group of Czech scientists proposed and verified a model in which an efficient function of bilirubin oxidation cycle required serum albumins as the matrix for biochemical conversions [50]. Their studies support the theory of important antioxidant role of bilirubin/biliverdin cycle, but situate it in plasma rather than inside of cells.

Interestingly, the beneficial effect of bilirubin is not limited to antioxidant properties. There is evidence that bilirubin can decrease production of proinflammatory cytokines such as TNF and IL-1*β* as well as downregulate Toll-like receptors 4 (TLR4) and inhibit expression of MyD88 as an adapter in IL-1 signal transduction [51].

Endothelial cells are capable of expressing on their surface molecules of major histocompatibility complex class II (MHC-II) in response to stimulation. Bilirubin was found to inhibit MHC-II expression through interference with the activation of signal transduction dependent on the signal transducer and activator of transcription-1 (STAT-1). Later study revealed that bilirubin reduces MHC-II also in dendritic cells and macrophages [52].

Bilirubin suppresses T cell proliferative response in the concentration physiologic for neonates (1.2-8.8 mg/dl) [52]. Further increase of bilirubin concentration above 8.8 mg/dl leads to T cell apoptosis. Moreover, bilirubin prevents oxidant-induced leukocyte adhesion in microvessels [53].

Bilirubin metabolism is also associated with nitric oxide (NO) metabolism. NO is a multifunctional gaseous molecule with free radical properties, playing role in numerous signaling pathways. It is produced by three isoforms of NO synthases (NOS): neuronal NOS (nNOS), endothelial NOS (eNOS), and inducible NOS (iNOS). During endotoxemia, bilirubin inhibits the induction of iNOS by bacterial lipopolysaccharide (LPS) and therefore alleviates tissue injury [54].

Summarizing, bilirubin might bring to neonate multidimensional benefits which are still not fully understood. Considering previous studies, it seems very likely that the increase in bilirubin concentration in the blood in the perinatal period is not accidental and contributes to maintaining homeostasis.

## 4. Carbon Monoxide: Another Relevant Player

CO used to be another underestimated product of heme degradation. It is infamous for its ability to bind to Hb over 200 times tighter than oxygen and formation of carboxyhemoglobin (COHb). The association is reversible but it reduces amount of available Hb molecules and can cause an impaired oxygen delivery to tissues. At least 86% of endogenous CO production comes from heme metabolism and remaining 14% derives from other processes such as lipid oxidation and metabolism of xenobiotics [25, 55]. CO is produced locally and does not result in systemic intoxication. Under basal conditions, HO-1 expression is generally low, except for cells involved in degradation of erythrocytes, and HO-2 is the main source of CO in most other tissues. Stress stimulus upregulates inducible HO-1 and increases significantly the amount of CO [25]. Blood COHb is a parameter of reference to assess the amount of endogenous or externally derived CO. Normal concentration of COHb in umbilical cord blood of newborns of nonsmoking mothers was estimated as less 1.2% [56]. Higher concentration might indicate that the mother smoked during pregnancy. COHb value rises in a neonate's blood during the first days after delivery and partially correlates with total serum bilirubin concentration, especially for values below 15 mg/dl [57].

Apart from Hb, CO ligates with heme moiety of soluble guanylyl cyclase (sGC), cytochrome p-450 (CYP-450), cytochrome-*c* oxidase (CcO), inducible nitric oxide synthase (iNOS), NADPH oxidases (NOX), and other cytochromes [25]. Numerous studies revealed important putative functions of CO. In vascular tissue [58] and the brain [59], CO produced constitutively by HO-2 is an important activator of sGC. CO participates via cyclic guanosine monophosphate (cGMP) in neurotransmission, regulation of vascular tone, inhibition of vascular smooth muscle proliferation, and platelet aggregation [59–62].

CO relaxes vascular smooth muscles also by direct activation of large conductance calcium-activated potassium channels (BK_Ca_) which is the most widely studied channel in the context of CO regulation [25, 63]. BK_Ca_ channels are found, i.e., in carotid bodies, where they play a central role in a response to hypoxia. Activity of several other channels was found to be modulated indirectly by CO by means of NO, cGMP, or ROS production [25]. ROS appears as a result of chemical asphyxiation of a cell when CO inhibits oxidase c (COX, complex IV of mitochondrium). CO binds to COX with high affinity, and a very little concentration of this gas is required to promote generation of ROS [25].

On the other hand, CO boosts antioxidative response as it activates a transcription factor NF-E2-related factor-2 (Nrf-2) [64]. Nrf-2 upregulates expression of HO-1, various ROS-detoxifying enzymes, and proteins, e.g., glutathione reductase (GR) GP-2, NADPH-quinone oxidoreductase (NQO), and light and heavy chains of ferritin complex [65].

Anti-inflammatory effect of CO is conferred through activation of the mitogen-activated protein kinase (p38 MAPK) pathway [66, 67], downregulation of the c-Jun N-terminal protein kinases (JNK) pathway [68], and the ERK1/2 extracellular signal-regulated kinases 1 and 2 (ERK1/2) [60]. Several studies focused on NOD, leucine-rich region, and pyrin domain containing 3 (NLRP3) inflammasome which promote maturation and release of proinflammatory cytokines. Different CO-releasing molecules or exposure to inhaled CO suppressed NLRP3 inflammasome [69–71], possibly by inducing pyrin production which is its negative regulator [72]. As the consequence, activation of caspase-1 was inhibited, concentration of IL-10 was increased, and IL-1*β* and IL-18 were decreased.

A modulation of caveolin-1-TLR4 interactions at the plasma membrane results in downregulation of Toll-like receptor 4 (TLR4) [73]. Decreased expression of complex consisting of TLR4 and myeloid differentiation factor-2 (MD-2) on the surface of dendritic cells and neutrophils during an immune response to endotoxin was also accredited to CO treatment [74, 75]. CO-treated mice manifest increased systemic tolerance and are less susceptible to endotoxic shock. Moreover, there were studies on baboons indicating that inhaled CO can speed up resolution of inflammation by increasing of lipoxin and eicosapentaenoic acid- (EPA-) derived E-series resolvins (RvE) synthesis [75, 76].

Taking into account all above facts, we can presume that CO, which production in the perinatal period rises proportionally to the production of bilirubin, might be another important modulator of immune response as well as contribute to achievement of oxidant-antioxidant balance in newborns.

## 5. Neonatal Diseases Related to ROS and Intense Inflammation

Reactive species are free radicals and substances which readily lead to free radical formation. They play important role in physiological processes such as maturation, cell signaling, or immune reactions. However, due to impaired electrons on their outer shell, free radicals react easily with DNA, proteins, and phospholipids causing their modification and loss of original functions. When antioxidant defense is insufficient, oxygen (ROS) and nitrogen (RNS) reactive species cause oxidative and nitrosative stress and therefore induce significant damage in the organism. During labor, a newborn changes the environment from intrauterine to extrauterine, and at the same time, partial oxygen pressure in newborn's arterial blood rises rapidly from 25 mmHg to 100 mmHg. As a result of hyperoxia, a significant amount of free radicals is generated [77, 78]. Among procedures which are common in neonatal intensive care units (NICU), oxygen therapy [79] and parenteral nutrition [80] markedly promoted oxidative damage. Nowadays, ROS/RNS are considered an important contributory factor in pathogenesis of neonatological diseases, i.e., retinopathy of prematurity (ROP), respiratory distress syndrome (RDS), bronchopulmonary dysplasia (BPD), periventricular leukomalacia (PVL), necrotizing enterocolitis (NEC), patent ductus arteriosus (PDA), intrauterine growth restriction (IUGR), and some congenital malformations [81].

### 5.1. Brain Injury

Brain injuries which frequently affect newborns are hypoxic-ischemic encephalopathy (HIE), intraventricular hemorrhage (IVH), and periventricular leukomalacia (PVL). The still-developing nervous system is very sensitive to any disturbances. The complex processes such as neuronal cell differentiation, migration, formation of synapses, and myelination can be easily disrupted by insufficient energy supply or accumulation of noxious compounds. Inflammatory and infectious processes pose a significant threat to nervous tissue by activation of various biochemical cascades which disturb brain metabolism. Oxidative stress in neonates triggers degeneration of vulnerable oligodendrocyte precursor cells, which leads to PVL [82]. White matter of immature myelin is susceptible to free radical damage because of high concentration of polyunsaturated fatty acids which easily undergo peroxidation and themselves become a source of new free radicals [83]. Increased lipid peroxidation follows episodes of acute hypoxia. Two NO synthases are upregulated by hypoxia episode: nNOS and eNOS. eNOS seems to have protective properties while nNOS activity might be harmful [83]. Glutamate is an important neurotransmitter but in high concentration can impair glutathione production by competitive inhibition of cystine uptake and as the result causes oxidative stress-mediated neuronal death [84]. In the neonatal brain, a temporary upregulation of glutamate receptors has been observed and it can also contribute to brain damage (excitotoxicity) [83].

It is certain that severe hyperbilirubinemia is dangerous to the infant's nervous system. Unconjugated bilirubin (UCB), as a lipid-soluble compound, crosses easily blood-brain barrier (BBB). When neurotoxic effect of bilirubin cannot be longer compensated by neuroprotective mechanisms, bilirubin-induced neurologic dysfunction (BIND) occurs in various parts of the brain including the basal ganglia, central and peripheral auditory pathways, and hippocampus [85]. The intensity of bilirubin neurotoxicity depends on several factors, e.g., UCB level, duration of hyperbilirubinemia, concentration of serum albumin, plasma pH, and BBB permeability. Proposed mechanism of bilirubin-induced neurotoxicity includes excessive release of glutamate, energy failure, and proinflammatory cytokine induction [38]. Despite the negative effect of bilirubin on brain cells, in vitro studies have shown neuroprotective role of bilirubin formed from constitutive expressed HO-2 within hippocampal and cortical neuronal cultures, when bilirubin occurred in nanomolar concentration [47]. Additionally, HO-2-derived CO is required for physiological functions in neuronal population [86]. Normal expression of HO-1 in neurons is low and resistant to induction. In contrast, astrocytes were able to increase their HO-1 expression by 7-fold within 3 h after exposure to hydrogen peroxide and they were less vulnerable to oxidative stress [87].

### 5.2. Pulmonary Dysfunction

Respiratory distress syndrome (RDS) occurs in 4-7% of all neonates. In term newborns, RDS is mostly caused by transient tachypnea of the newborn and pneumonia, less frequently by meconium aspiration syndrome and congenital respiratory system defects [88]. Preterm infants develop RDS due to immature lungs and insufficient or dysfunctional surfactant. Without functional surfactant, alveoli collapse upon expiration. Mechanical ventilation (MV) with increased mean airway pressure is often necessary to maintain adequate relationship of ventilation and perfusion, and to prevent respiratory failure. Therefore, preterm neonates are additionally exposed to hyperoxia, which activates NOX to intensive ROS production and promotes destruction of alveoli endothelial cells and release of proinflammatory cytokines [81, 89]. MV induces oxidative stress and leads to further lung injury [90].

A rising number of bronchopulmonary dysplasia (BPD) cases are recorded every year, although its definition has changed since it was first described in 1967. The clue of BPD pathogenesis is airways remodeling as the result of chronic inflammation and impairment of alveolar epithelial type II cells. Those important stem cells synthesize surfactant, control transepithelial movement of water, and regulate lung tissue development and regeneration. Hyperoxia induces death of alveolar epithelium and vascular endothelium. Loss of integrity of vascular cells results in edema and increased inflammatory cell migration to the lung tissue. Due to stromal cell proliferation and depleted vascularization of distal lung tissues, children with BPD may develop pulmonary hypertension [91].

Studies in mice confirmed that in the neonatal lung, HO-2 represents an important antioxidative mechanism. In HO-2 knockout mice, parameters of oxidative stress after hypoxia were higher and their ferritin level was insufficiently increased in relation to increased iron content, which led to accumulation of redox-active iron and exacerbation of oxidative stress injury [92].

Effects of HO-1 activity are complex and depend not only on its expression level but also on duration of HO-1 expression and its subcellular localization. This protein is the most abundant in the smooth endoplastic reticulum, anchored to its c-terminus, but it is also found in other cell compartments. Induction of mitochondrial HO-1 improves energy metabolism and prevents drop of ATP level [93]. Nuclear HO-1 upregulates genes protecting against oxidative stress and this action is independent from enzymatic activity [94].

In newborn rodents, HO-1 expression is higher than in adults but it is less susceptible to further induction, probably due to enhanced expression of Bach1 - nuclear repressor of HO-1 transcription-Bach1 [95]. HO-1-deficient mice presented disrupted alveolar growth [96]. However, HO-1 overexpression is also undesirable since it results in maladaptive proliferation of epithelial type II cells and impaired lung function [97].

Carbon monoxide—one of the products of HO reaction—plays an important role in defence against oxidative lung damage. The protective role of low doses of CO in hyperoxia-induced lung injury was discovered in a rodent model. CO at the concentration of 250 ppm (0.025%) prevented development of pulmonary injury manifestations (pleural infusion, protein accumulation, lung hemorrhage, edema, alveolar septal thickening, influx of inflammatory cells, and fibrin deposition) and prolonged the survival time of animals exposed to lethal hyperoxia [98]. In endothelial cells, CO exerts antiapoptotic activity through mechanism involving activation of the p38 MAPK and NF-*κ*B pathways [67, 99, 100].

### 5.3. Retinopathy of Prematurity (ROP)

The eye is an organ which at the early stage of life is highly susceptible to changes in oxygen concentration. Vascularization begins in a 16-17-week-old fetus and is stimulated by various hormonal factors as well as “physiological hypoxia” [101]. Intense exposure to oxygen causes loss of vessels at the first stage, followed by neovascularization at the next stage. Pathological vessels are sources of various ophthalmological problems including retinal hemorrhages, retinal detachment, and intravitreal neovascularization. Moreover, hyperoxia induces abundant retinal mitochondria to ROS overproduction, which further contributes to ROP progression. Results of multicenter trial with recombinant human superoxide dismutase (rhSOD) seem to confirm the role of oxidative stress in ROP pathology. Infants treated with rhSOD were less likely to suffer from severe ROP and they less frequently develop severe amblyopia or complete blindness [102]. Also, intramuscular injection of vitamin A, a recognized antioxidant, improves retinal function and decreases risk of eyesight loss [103].

The impact of bilirubin on ROP incidence and severity remains undetermined. Some studies claimed a reverse correlation between mean bilirubin level [104] and peak bilirubin level [105] in the first 2 weeks of life and the severity of ROP [104], while other studies denied protective effect of bilirubin on the development of ROP [106–108].

### 5.4. Necrotizing Enterocolitis

Like in the case of the previously described diseases, the main risk factor of NEC is prematurity. Feeding and bacterial colonization activate a defective immune response in the immature intestinal system leading to perfusion dysregulation and uncontrolled inflammation [109]. Large quantities of platelet-activating factor (PAF), TNF, and IL-6 are released and boost migration of leukocytes to the damaged tissue. Polymorphonuclear leukocytes are important producers of free radicals. Ischemia-reperfusion episodes also trigger increased ROS production resulting in mucosal injury [110]. An ischemia-reperfusion injury of gut tissue was induced in rats. Animals which were simultaneously treated with a continuous infusion of bilirubin presented less histopathologic and biochemical evidence of damage than the untreated group [111]. Moreover, in an experiment on a mouse model, heterozygous animals with partially deficient HO-1 (Hmox1(+/-)) were more susceptible to experimental NEC-like intestinal injury than a wild type [112], which additionally points out the important role of oxidative stress in NEC pathogenesis. In terms of human observational studies, significantly lower mean total serum bilirubin was found in preterm neonates with NEC in comparison with healthy newborns [104].

## 6. Clinical Opportunities regarding Elements of Heme Catabolism

Antioxidant and anti-inflammatory properties of bilirubin and CO as well as the beneficial effect of heme oxygenase (HO) upregulation have become a focus of interest for researchers looking for new ways of treatment. Earlier reports about amelioration of a disease course during jaundice, observational studies of patients with Gilbert syndrome, and experiments on animal models provide us with some hints about in which illness heme-derived compounds can be effective. Based on the previous observations, it can be concluded that such a treatment would be able to improve endothelial function, reduce oxidative stress, and mitigate inflammatory response; therefore, it might be efficient in the case of cardiovascular diseases [113–115], diabetes mellitus 2 [116–119], inflammatory bowel diseases [51, 120–122], transplant rejection [123, 124], sepsis [64, 69, 70], rheumatic diseases [125, 126], wound healing [127, 128], ischemic-reperfusion injuries [129–131], and others.

One way to increase the quantity of heme catabolism products in an organism is upregulation of HO-1. This approach was adopted in numerous studies [23, 26, 47, 53]; upregulation of heme oxygenase can be obtained either by administration of HO inducers or gene transfer [26, 132].

Another way to increase the quantity of heme catabolism products is supplementation. A large part of our knowledge about bilirubin, biliverdin, and CO properties come from testing those substances on animal models, mainly via intravenous administration in the case of bilirubin and via inhalation in the case of CO. Bilirubin was also administrated to healthy human volunteers without causing evident adverse effects [133]. Many difficulties concerning the application of native substances may appear, including water insolubility and potential neurotoxicity of bilirubin or high affinity of CO to Hb. However, new technologies come in handy and enable us to overcome some of those limitations.

In 2016, a new bilirubin compound was synthetized. Bilirubin molecules with covalently attached polyethylene glycol (PEG) tend to aggregate spontaneously and form bilirubin nanoparticles (BRNPs). BRNPs have diameter of approximately 100 nm, dissolve in water, and display valuable properties of bilirubin without causing jaundice [134, 135]. BRNPs showed preferred accumulation at the inflamed tissue and longer circulation time, which positively influences its efficacy as a hydrogen peroxide scavenger [135]. The first study which tested newly invented BRNP indicated that BRNPs-treated mice were protected from dextran sodium sulfate-induced colitis [134]. Clinically, they manifested no intestinal bleeding or diarrhea; histologically, there was little immune cell infiltration in mucosa, submucosa, and muscle layers of the intestines compared to untreated mice. In the experiment on a mouse model of allergic asthma, BRNPs turned out to be clearly more active than unconjugated bilirubin and more effectively reduced population of activated Th2 cells as well as alleviated airway hyperresponsiveness [135]. Bilirubin nanoparticles were also found to enhance and prolong graft pancreatic islet survival and seem to be a very promising treatment against transplant rejections [136].

CO is another advantageous molecule which can be applied in treatment of various diseases. Delivery of gaseous CO to the target tissue is practically impossible because of the lack of specificity and high affinity to Hb. In order to make use of CO anti-inflammatory and cytoprotective properties, a nontoxic CO-releasing agent is required. Such a molecule should also be biocompatible and easy to mobilize. Most of CO-releasing molecules (CORMs) are organometallic compounds which include carbonyl complex with transient metal core. In recent years, some nonmetallic CORMs [137] have also been invented. Lower toxicity and easier modification may be in favor of the nonmetallic CORMs. Regardless of CORM type, two parts can be distinguished—a CORM and a drug sphere. The former is responsible for the mechanism of CO discharge and the number of released CO molecules; the latter determinates additional properties of CORM which gives it an advantage over inhaled CO, especially the ability to target the desired tissue [138].

Several different kinds of CO-releasing molecules have been already tested on animal models.

The list of potential therapeutic applications, including a vast range of diseases and pathological conditions, has been gathered in the table (Table 1).

Up to date, there were no studies focusing on the treatment of neonatal diseases with the use of either BRNPs or CORM technologies. In fact, none of these therapeutic strategies has been verified in clinical trials. However, reports concerning ameliorative activity of these substances in colitis, brain, or lung injuries in animal models seem promising. It should be considered that in spite of relatively high concentration of bilirubin in newborn's blood, which even put them in danger of kernicterus, neonates may still benefit from bilirubin or CO administration. At this group of patients' simple parenteral administration of bilirubin or induction of HO-1 would be undesirable, but there is definitely a need for antioxidant and anti-inflammatory treatment. In the case of preterm infants, the new molecules might be of great use as they display higher efficiency with lower toxicity. There is a good example of bilirubin nanoparticles which act as free radical scavengers and support antioxidant defense without generating jaundice.

## 7. Neonatal Jaundice: Different Therapeutic Approaches

Having discussed the role of heme catabolism in newborns, it is worth addressing again the subject of neonatal jaundice. This condition occurs in almost 2 out of 3 term infants as the result of disproportion between increased bilirubin production and less effective elimination of the pigment. Prematurity, hematomas, glucose intolerance in pregnancy, hemolysis, and mutations in a UGT gene are additional well-known factors which intensify bilirubin formation and boost the probability of jaundice-related complications [139]. In many cases, medical interventions are required to prevent bilirubin encephalopathy. A detailed description of the pathogenesis and consequences of neonatal jaundice is not the subject of this review. However, we would like to highlight a few aspects of jaundice treatment concerning safety of phototherapy and possible application of HO inhibition strategy.

Phototherapy of jaundiced neonates is a recognized and efficient method of serum bilirubin level reduction. The wavelength around 460 nm is considered safe and efficient because of good light penetration into the skin. Upon radiation exposure, a naturally occurring insoluble Z, Z-bilirubin changes into water-soluble configurational photoisomers Z, E-bilirubin and E, Z-bilirubin, and into structural photoisomers Z- and E-lumirubin [140]. Only small amounts of lumirubin can be detected in an infant's body as it is quickly excreted into the urine and stool. The configurational isomers are eliminated more slowly and they can be reversed back to Z, Z-bilirubin. Importantly, bilirubin photoisomers can be removed from the organism without earlier glucuronidation. It is believed that Z, E- and E, Z-bilirubins are incapable of crossing the blood-brain barrier and therefore do not produce neurotoxic effects. However, more studies are desirable to confirm this hypothesis [141, 142]. There is also a need for thorough investigation of bilirubin photoisomers toxicity, especially in the context of aggressive phototherapy of very-low-body-weight infants [141, 143]. Moreover, Stevenson et al. raised question of oxidative stress resulting from bilirubin and riboflavin photosensitization [139].

Overheating, dehydration, hypocalcemia, conjunctivitis, and retinal damage are adverse effects observed after phototherapy [141, 144]. Additionally, neonates with porphyrinemia due to hepatic dysfunction or intensive hemolysis are in the risk of developing purpuric or bullous eruption and “bronze” baby syndrome. Brown skin pigmentation and purpuric eruptions are benign complications which resolve within few days after cessation of the phototherapy [144, 145].

Metalloporphyrins (Mps) are synthetic heme analogues which bring a promise of specific medical intervention for prevention and treatment of neonatal jaundice. Mps compete with heme for binding with the heme oxygenase; therefore, they decrease bilirubin production. Two kinds of Mps have been already studied in human clinical trials—tin protoporphyrin (SnPP) and tin mesoporphyrin (SnMP). SnPP was abandoned because of prominent photosensitizing properties [146]. SnMP proved efficient in reducing plasma peak bilirubin and the need for phototherapy [147, 148]. Nevertheless, some neonates still required phototherapy. In this group, a transient erythema was recorded as the only short-term side effect and occurred after exposure to white light. Some other Mps, such as chromium mesoporphyrin, zinc protoporphyrin, and zinc bis glycol, possess desirable properties. They are photoinert, can be administrated orally (SnMP requires intramuscular injections), and affect other heme-dependent enzymes in a lesser degree than SnMP [146, 149].

In the light of beneficial functions of bilirubin and CO, which are meticulously presented in this paper, additional questions might arise—What is the impact of phototherapy and Mps application on natural defensive strategies of a neonate against oxidative stress and inflammatory processes? Is the reduction of bilirubin encephalopathy occurrence the only goal to obtain or should we also take into account other aspects such as occurrence of ROS-related diseases in premature babies? The paper does not try to answer these questions and only points out at future challenges.

## 8. Conclusions

Summing up, the role of heme metabolism products is more multifaceted than it was assumed in the past. They modulate inflammation and ameliorate generation of noxious free radicals. As it was presented above, aggravated inflammatory reactions and increased oxidative stress are often implicated in the pathology of typical neonatal diseases. The more premature a newborn is, the higher is his susceptibility to oxidative stress and the greater risk of serious illnesses. Increased HO-1 activity and high bilirubin concentration are the response to stress stimuli faced by infants and constitute an important part of neonatal protection. In the case of jaundice of the newborn, its positive aspects should be taken into account and bilirubin-lowering therapy should be based on clinical indications and careful risk assessment of hyperbilirubinemia complications. Newly developed bilirubin nanoparticles and CO-releasing molecules show good effects in the studies on animal models of inflammatory diseases. Moreover, they are characterized by lower toxicity and better controllability. There is a hope that in future some of those molecules can be employed in prevention and treatment of neonatal diseases connected with increased oxidative stress and an excessive inflammatory reaction.

## Figures and Tables

**Table 1:** Examples of CO-releasing molecules (CORMs) and their clinical applications. The table was based on Ismailova et al. [138].

Molecule	Clinical applications
CORM-2	Antibacterial activity (*E. coli*^∗^, *H. pylori*^∗^, *P. aeruginosa*^∗^), neuroprotection^∗∗^, cochlear inflammation^∗∗^, neuropathic pain^∗∗^, colitis^∗∗^, bacterial LPS-induced inflammation^∗∗^, TNF-*α*-induced inflammation^∗^, inflammation-induced blood clotting^∗^, abnormal platelet coagulation^∗^, intestinal mucosa injury^∗∗^, sepsis^∗∗^, hyperglycemia^∗∗^, obesity^∗∗^, cancer (prolonged survival^∗∗^, decreased angiogenesis^∗∗^, and cell aggregation^∗^), cardioprotection^∗^, kidney transplantation^∗∗^
CORM-3	Antibacterial effect^∗^ (*H. pylori*^∗^, *S. typhimurium*^∗^, *P. aeruginosa*^∗∗^), neuroinflammation^∗^, periodontal inflammation^∗^, septic lung injury^∗∗^, cardioprotection^∗∗^, hemorrhagic shock^∗^, cardiac transplantation^∗∗^, renoprotection^∗^, postoperative ileus^∗∗^, increased intraocular pressure^∗∗^, anticoagulation^∗∗^, vascular inflammation^∗^, pulmonary hypertension^∗∗^
PhotoCORM/TryptoCORM	Antibacterial effect against *E. coli*^∗^, *N. gonorrhoeae*^∗^
CORM-371	Antibacterial effect against *P. aeruginosa*^∗^
CORM-A1	Antibacterial effect against *P. aeruginosa*^∗^, improved neurodifferentiation^∗^, diabetes (facilitated beta cell regeneration)^∗∗^, obesity^∗∗^, autoimmune uveoretinitis^∗∗^, hemorrhagic shock^∗∗^, liver injury^∗∗^, anticoagulation^∗∗^
ALF186, ALF492	Neuroprotection (ischemic insult^∗^, malaria^∗∗^)
CORM-401	Efficient vasodilator^∗^
CO-Hbv	Colitis^∗∗^

^∗^In vitro studies (cell/tissue cultures). ^∗∗^In vivo studies (rodent models).
